# MAPK8 and HDAC6: potential biomarkers related to autophagy in diabetic retinopathy based on bioinformatics analysis

**DOI:** 10.3389/fendo.2025.1487007

**Published:** 2025-05-21

**Authors:** Ruotong Sun, Ling Zuo

**Affiliations:** Department of Ophthalmology, The Second Norman Bethune Hospital of Jilin University, Changchun, China

**Keywords:** diabetic retinopathy, autophagy, biomarker, MAPK8, HDAC6

## Abstract

**Introduction:**

One of the most common vascular diseases of the retina is diabetic retinopathy (DR), a microvascular condition caused by diabetes. The autophagy system transports and degrades cytoplasmic substances to lysosomes as part of the intracellular degradation process. Autophagy appears to be an important regulator in the development and progression of DR, but its mechanism and potential role are unclear. The purpose of this study is to identify autophagy-related genes in DR and find potential biomarkers associated with DR through bioinformatics analysis.

**Method:**

We retrieved the dataset GSE102485 from the Gene Expression Omnibus (GEO) database and compiled a list of 344 autophagy-related genes. Using the R software, bioinformatics analysis was used to identify the differentially expressed autophagy-related genes (ARGs). Then, we identified the autophagy-related hub genes (ARHGs) through a series of analyses including Gene Ontology (GO) enrichment analysis, Kyoto Encyclopedia of Genes and Genomes (KEGG) pathway analysis, correlation analysis, and protein-protein interaction (PPI) network. In addition, the miRNA-gene-TF interaction network was generated using the NetworkAnalyst platform. Potential therapeutic drugs were predicted utilizing the Drug-Gene Interaction Database (DGIdb). Ultimately, DR was simulated through the high glucose incubation of the retinal pigment epithelium cell line (ARPE-19), and employing quantitative real-time polymerase chain reaction (qRT-PCR) to verify ARHG expression. The effectiveness of ARHGs in diagnosing DR was assessed by measuring the area under the receiver operating characteristic (ROC) curve.

**Results:**

Differential expression analysis identified 26 ARGs, of which 6 were upregulated and 20 were downregulated. Through GO and KEGG enrichment analysis, it was found that ARGs showed significant enrichment in autophagy-related pathways. Using PPI network analysis, 7 ARHGs were identified. The expression of MAPK8, HDAC6, DNAJB1 and TARDBP, in a model of DR were confirmed by qRT-PCR. The ROC curve results showed that MAPK8, HDAC6, DNAJB1 and TSC2 had high predictive accuracy and could be used as biomarkers for DR.

**Conclusion:**

Through bioinformatics analysis, we identified 26 genes that may be associated with autophagy in DR. We suggest that the hub genes MAPK8 and HDAC6 as biomarkers may be involved in autophagy in DR.

## 1 Introduction

Diabetic retinopathy (DR) ranks as a prevalent microvascular disease associated with diabetes. It is also the leading cause of blindness in adults. Research forecasts that by 2030, the diabetic population might ascend to 552 million, with over a third exhibiting symptoms of DR (1). DR manifests as a range of pathological alterations, including thickening of the vascular basement membrane, impaired blood-retinal barrier function, capillary cell death, cell-free capillary formation, neovascularization, and retinal detachment (2). Many molecular mechanisms have been proposed to explain the development of DR, including oxidative stress, formation of advanced glycation end products, activation of protein kinase C and polyol accumulation (3). Such processes enhance the advancement of diseases through alterations in cellular signaling and cell metabolism, which in turn lead to high production of inflammatory factors and vascular endothelial growth factor (VEGF). Notably, VEGF is known to be an important target for the treatment of DR, age-related macular degeneration and macular edema (4). Currently, the main treatments for DR are intravitreal injections of anti-VEGF agents, total retinal laser photocoagulation, and vitrectomy. For patient’s refractory to anti-VEGF agents, intravitreal corticosteroid injections and macular laser treatment may also be considered. However, there are still a large number of patients with refractory DR, and these approaches may not be able to fully prevent and treat DR, and the exact pathogenesis and signaling pathways behind the disease are not yet fully understood (2). Therefore, further research on the pathogenesis of DR at the molecular level and the discovery of new biomarkers are crucial for patient prognosis and quality of life improvement.

Autophagy is a specialized catabolic pathway that maintains homeostasis by degrading damaged organelles, abnormal proteins, and breaking down cellular components (5). Stimulated by various pathophysiological responses, such as hypoxia, growth factor removal, nutrient deprivation, or enhanced ROS release, leading to increased demand for intracellular nutrients and energy, the process of autophagy needs to be augmented to meet cellular demands (6). Recently, it has been shown that the autophagic process plays an important role in the pathogenesis of DR and that it can influence the progression of such diseases (7). Abnormal levels of autophagy contribute to the progression of DR, including processes that promote vascular endothelial cell injury, vascular endothelial growth factor release, neovascularization, and even retinal neuroepithelial injury (8, 9).

Retinal pigment epithelial (RPE) cells serve as part of the outer blood-retinal barrier (BRB), and their role is to maintain the normal structure of the retina and perform their own functions (10). The primary cause of vision loss in patients with DR is macular edema, and disruption of the blood-retinal barrier is the key to its occurrence (11). In diabetes, disruption of the outer BRB is associated with regulation of autophagy in RPE cells. *In vitro* studies have demonstrated that ARPE-19 cells exhibit an increase in autophagy in response to high glucose levels (12). However, the exact mechanism of how autophagy in RPE cells contributes to the progressive development of DR needs to be further explored.

Therefore, in this study, we analyzed datasets of patients with DR and non-diabetic individuals to reveal differentially expressed genes (DEGs) in the retina of patients with DR and controls. Subsequently, we matched these genes with autophagy-related genes to obtain differentially expressed autophagy-related genes (ARGs), and, finally, performed functional enrichment analysis to elucidate the their interactions and biological function. Immediately after, we screened for autophagy-related hub genes (ARHGs) through protein-protein interaction (PPI) network analysis and further confirmed with qRT-PCR in the DR model. In addition, we constructed an miRNA-gene-TF regulatory network and predicted potential targets for pharmacotherapy based on ARHGs.

In summary, our results further reveal the role of autophagy process in DR and its mechanism, and these ARHGs lay the foundation for future studies of DR at the molecular biological level, as well as provides a valid biomarker for its use in the diagnosis and treatment of DR.

## 2 Materials and methods

### 2.1 Data acquisition and preprocessing

**Figure 1:**
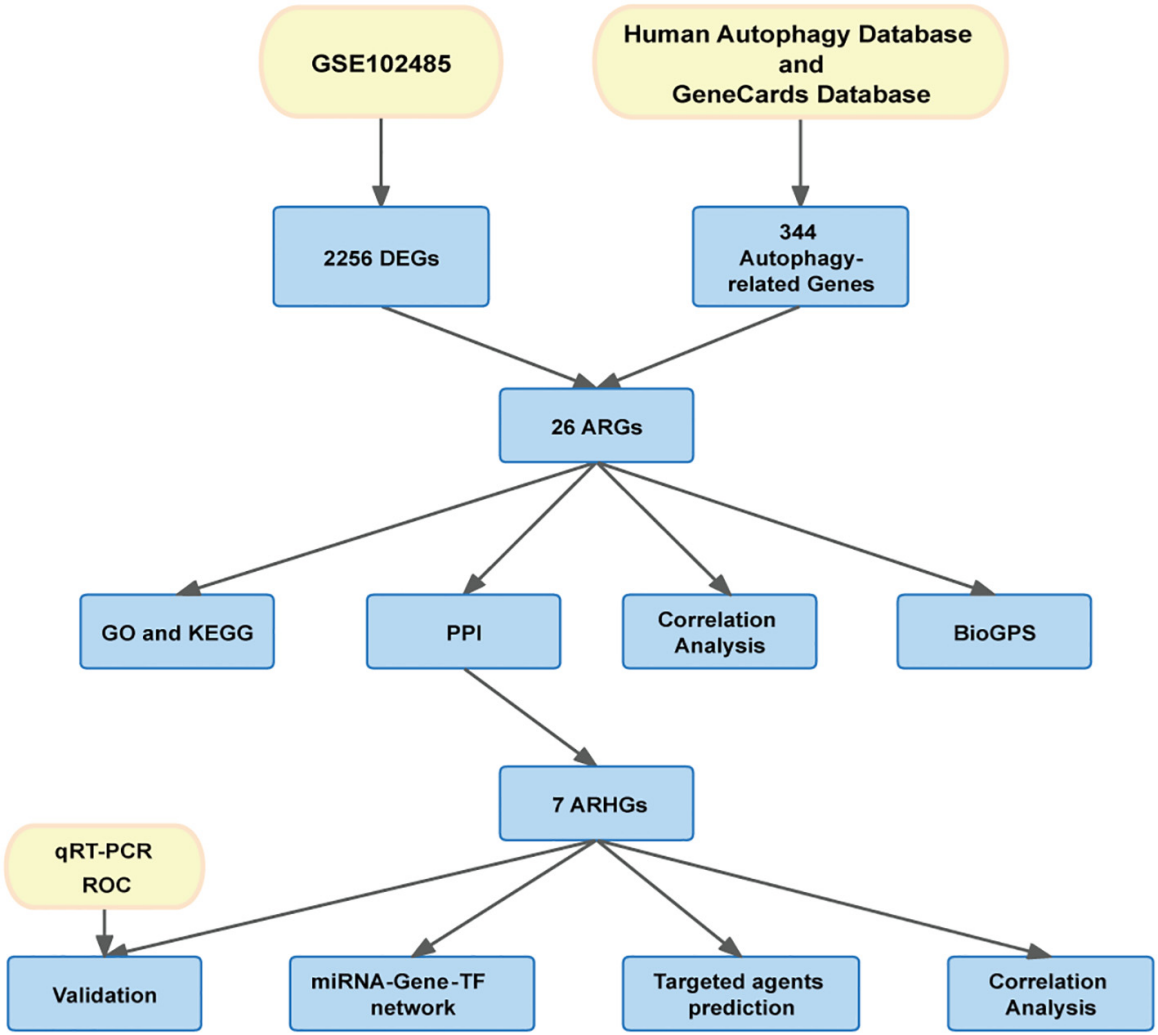
Overview of the research procedure of this study.

The flow chart of this study is shown in **Figure 1**. Download GSE102485 by Li Y et al. (13) from the Gene Expression Omnibus (GEO) database (14) (http://www.ncbi.nlm.nih.gov/geo/). GSE102485 was in GPL18573 platform. The GSE102485 dataset was selected for its unique focus on human retinal tissues, which provides direct insights into DR-related molecular alterations in the ocular microenvironment. The dataset included 3 normal retina samples which were collected in 3 healthy controls, 2 retinal periphlebitis samples, 3 branch retinal vein occlusion samples, and 22 DR samples (DR group). DR and control group data were retained in this study for analysis. We collected autophagy-related genes from different databases. The Gene Cards database (15) (https://www.genecards.org/): We used the terms “autophagy” as search keywords, and only retained autophagy-related genes with relevance score > 5.000 and “protein coding”tag. A total of 210 autophagy-related genes were retrieved from the Gene Cards database. The Human Autophagy Database (http://www.autophagy.lu/): We obtained 232 autophagy-related genes from The Human Autophagy Database. Finally, a total of 344 autophagy-related genes were acquired after merging and deleting duplicates.

### 2.2 Differential expression analysis

We used R software (version 4.3.2) for data quality control, background correction, standardization, and subsequent analysis. The “limma” package (16) was used to screen for differential genes between the control and DR groups. We considered genes with adjusted p-values <0.05 and |log fold change|>1 as differentially expressed genes. Next, we overlapped DEGs with autophagy-related genes to obtain ARGs. To visualize the differential genes, the “ggplot2” (17) “heatmap” (18) and “ggvenn” packages(https://github.com/yanlinlin82/ggvenn) were employed to generate volcano, heatmap, and Venn diagrams, respectively.

### 2.3 Correlation analysis and tissue-specific expression

Correlation analysis of ARGs was performed using the Spearman correlation in the “corrplot” package. We used the BioGPS website (http://biogps.org) to study the tissue-specific expression of ARGs.

### 2.4 Functional enrichment analysis

We analyzed and evaluated the function of ARGs by Gene Ontology (GO) and Kyoto Encyclopedia of Genes and Genomes (KEGG) using the “clusterProfiler” software package (19). GO analysis included biological processes (BP), cellular components (CC) and molecular functions (MF) (20). KEGG analysis of possible pathways involved in ARGs (21).

### 2.5 Generation of a protein-protein interaction network and identification of hub genes

We constructed a PPI network of ARGs using the Search Tool for Interactive Gene Retrieval (STRING) (22) (https://cn.string-db.org/) to explain the interactions between ARGs. Visualize and build PPI networks with Cytoscape v3.9.1 software (23). The top 10 genes in the PPI network calculated based on the maximum clique centrality (MCC), degree, maximum neighbor component (MNC) and maximum neighbor component density (DMNC) methods in the cytohubba plug-in, respectively, and the overlapping genes were determined to be the hub genes.

### 2.6 miRNA-gene-TF interaction networks

The NetworkAnalyst 3.0 platform (https://www.networkanalyst.ca) was used to analyze the interactions between ARHGs, miRNAs, and TFs (24).The miRNA-gene-TF network was visualized by Cytoscape software.

### 2.7 Prediction of potential agents

The Drug-Gene Interaction Database (DGIdb) (https://dgidb.org/) (25) was used to predict potential therapeutic agents that target ARHGs. Using drug-gene interaction data, we can search for potential drugs that can target specific target genes.

### 2.8 Culturing cells to model DR

The human retinal pigment epithelial cell line (ARPE-19) was procured from Shanghai Zhong Qiao Xin Zhou Biotechnology in China (PRI-H-00004). The cells were cultured in Dulbecco’s Modified Eagle Medium/Nutrient Mixture F-12 (DMEM/F12) medium supplemented with 10% fetal bovine serum (FBS) (Zqxzbio, Shanghai, China) and 1% Antibiotic-Antimycotic (Zqxzbio, Shanghai, China) at 37°C with 5% carbon dioxide. ARPE-19 cells were cultured in high glucose medium containing 25 mM anhydrous glucose (Zqxzbio, Shanghai, China) for 72 h as an *in vitro* diabetes model (26–28), and ARPE-19 cells were cultured in normal medium containing 5 mM glucose for 72 h as a control group. The culture medium was changed every 2 days. When the cell density reached 80%, the cells were passaged at a ratio of 1:3, washed with PBS (Zqxzbio, Shanghai, China) and treated with 0.05% trypsin (Zqxzbio, Shanghai, China). In order to guarantee the precision and reliability of the findings, the study was conducted using 8-10 generations of cells. Cells were routinely screened for mycoplasma contamination using a probe-based qPCR assay (MeilunBio, MA0350).

### 2.9 qRT-PCR and validation

Total RNA was extracted from cells in the DR group and control group using the Total RNA Extraction Kit (Sevenbio, Beijing, China) according to the manufacturer’s protocol. A NanoDrop 2000c spectrophotometer (Thermo Fisher Scientific) was used to test the concentration and purity of RNA. Then, the total RNA was reverse transcribed with StarScript III RT kit (Genstar, Beijing, China) to obtain cDNA, and qRT-PCR was utilized with the StarScript III one-step qRT-PCR SYBR kit (Genstar, Beijing, China), the above steps were performed according to the manufacturer’s instructions. Sangon Biotech Co., Ltd (Shanghai, China) designed and synthesized the primers, and the primers sequences are shown in **Supplementary Table S1**. The relative expression level of the gene mRNA was normalized using β-actin (12) as the reference gene and the calculation of mRNA expression levels was performed using the 2-DDCt method. This *in vitro* experiment was repeated three times. The GSE221521 (29) dataset, obtained from the GEO database, contains 69 peripheral blood samples from patients with DR and 50 from healthy individuals. We used R software (version 4.3.2) for data quality control, background correction, and standardization of the GSE221521, and used the GSE221521 dataset to validate the diagnostic efficacy of the hub genes. We built the receiver operating characteristic (ROC) curve to assess the diagnostic significance of ARHGs using the pROC package (30) in R. The area under the ROC curve (AUROC) was used to represent the magnitude of diagnostic efficiency.

### 2.10 Statistical analysis

The statistical analysis in this study were executed through the utilization of the GraphPad Prism software (version 9.5.1) Normality of data distribution was confirmed using the Shapiro-Wilk test. All datasets met the normality assumption (P > 0.05). The analysis of gene expression levels within the sample was conducted employing the Student’s t-test, and the observed difference was deemed to be statistically significant when P <0.05.

## 3 Results

### 3.1 Identification of differentially expressed ARGs

**Figure 2:**
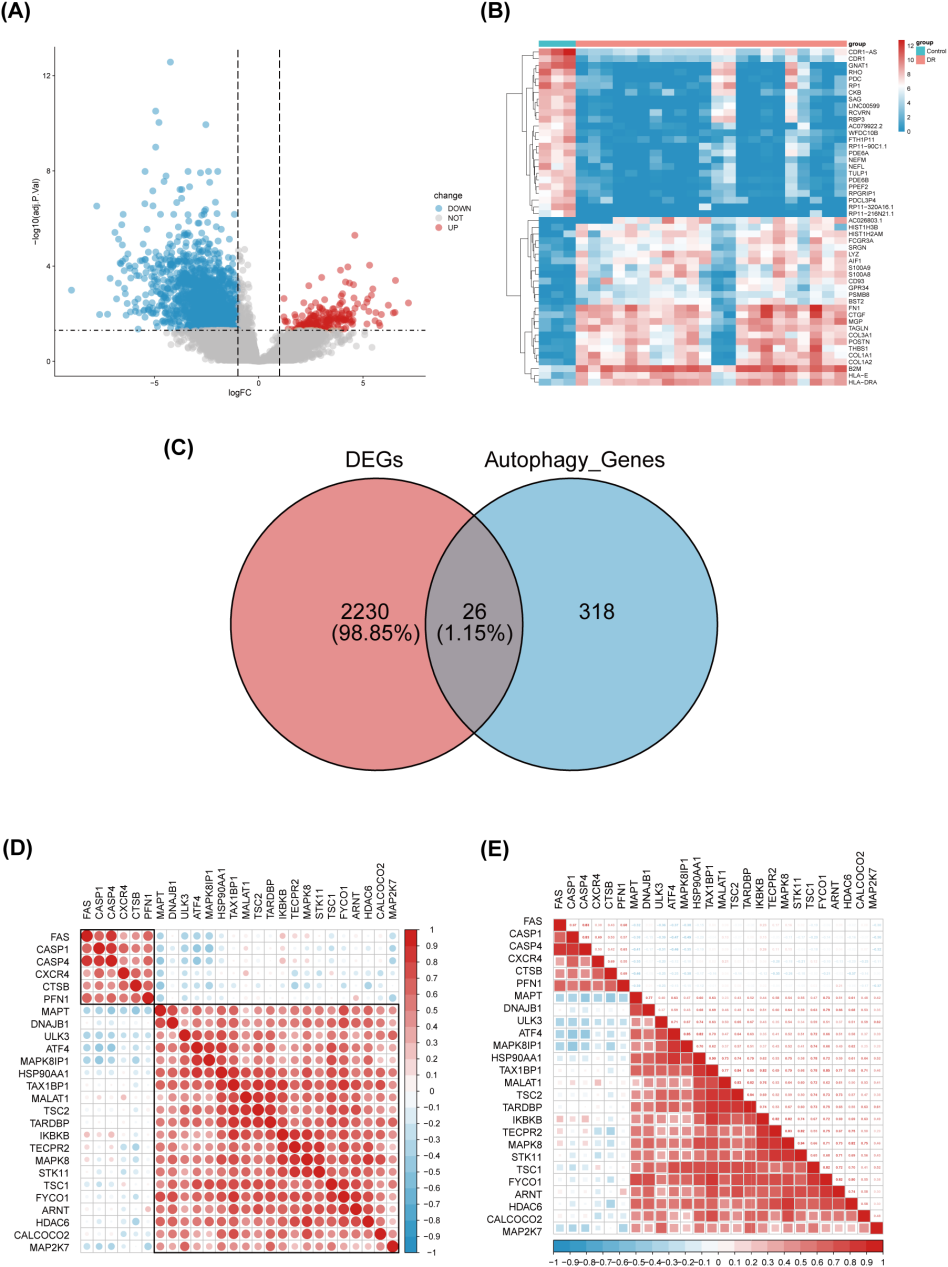
Identification of differentially expressed genes involved in autophagy in DRof GSE102485. **(A)** Volcano plot of 2256 differentially expressed genes in the GSE102485 dataset. It contains 2211 significantly up-regulated genes, represented by red dots, and 2045 significantly down-regulated genes, represented by blue dots, whereas gray dots represent stably expressed genes. **(B)** Heatmap of the top 50 significantly differentially expressed genes in the GSE102485 dataset. The blue bars represent control specimen, denoted by “Control”, and the red bars represent specimens from patients with DR, denoted by “DR”. **(C)** Venn showing 26 common genes between differentially expressed genes and autophagy-related genes. **(D, E)** 26 differentially expressed autophagy-related genes correlation heatmap. The color red is used to indicate a positive correlation, while the color blue is used to indicate a negative correlation.

Differential gene expression analysis was performed using gene expression information from the GSE102485 dataset, comprising 22 DR retinal tissue samples and 3 normal retinal tissue samples. A total of 2256 differentially expressed genes were identified, including 211 up-regulated genes and 2045 down-regulated genes, using the adjusted p-value < 0.05 and |logFC|>1 as criteria. Differential expression of these 2256 DEGs was plotted using volcanic chart and heatmap (**Figures 2A, B**). According to the GeneCards database and the Human Autophagy Database, a total of 344 genes involved in the process of autophagy were obtained (**Supplementary Table S2**). After comparing 2256 DEGs with 344 autophagy-related genes, 26 overlapping genes were identified (**Figure 2C**). Then, 26 ARGs in DR were obtained, including 6 upregulated genes and 20 downregulated genes (**Table 1**).

**Table 1:** The 26 differentially expressed autophagy-related genes in DRsamples compared to normal samples.

Gene Symbol	logFC	P-value	Adjusted P-value	Changes
*PFN1*	3.94968	0.0000243	0.0018	UP
*CXCR4*	3.60314	0.000138527	0.00627	UP
*CASP1*	3.22422	0.000499268	0.01551	UP
*CTSB*	3.19115	0.001586652	0.03497	UP
*CASP4*	2.91775	0.000110479	0.00529	UP
*FAS*	2.19346	0.001068984	0.02646	UP
*MAP2K7*	-1.33203	0.001440462	0.03272	DOWN
*STK11*	-1.35821	0.000508236	0.01568	DOWN
*TECPR2*	-1.38652	0.000813711	0.02178	DOWN
*MAPK8IP1*	-1.45568	0.000657198	0.01875	DOWN
*IKBKB*	-1.64286	0.002065839	0.04259	DOWN
*ARNT*	-1.72436	0.000644245	0.01851	DOWN
*ATF4*	-1.78547	0.001071365	0.02649	DOWN
*HDAC6*	-1.80076	0.001925497	0.04062	DOWN
*TARDBP*	-1.89154	0.002498207	0.04825	DOWN
*MAPK8*	-1.90305	0.000631113	0.01831	DOWN
*TAX1BP1*	-1.9412	0.0002422	0.00926	DOWN
*MAPT*	-1.99935	3.6E-10	6.27E-07	DOWN
*DNAJB1*	-2.14703	0.000906395	0.02352	DOWN
*TSC1*	-2.30514	0.0000587	0.00339	DOWN
*TSC2*	-2.337	0.00000437	0.00052	DOWN
*CALCOCO2*	-2.34208	0.000000244	7.50E-05	DOWN
*FYCO1*	-2.40975	0.0000135	0.00118	DOWN
*MALAT1*	-2.84857	0.001197985	0.02883	DOWN
*HSP90AA1*	-2.99295	0.0000334	0.00224	DOWN
*ULK3*	-3.2608	0.0000251	0.00184	DOWN

### 3.2 Correlation analysis and tissue-specific expression of ARGs

We performed a correlation analysis of 26 ARGs using bioinformatics methods with the aim of investigating the correlation between these genes. The analysis revealed a pronounced association between the up-regulated and down-regulated genes (**Figures 2D, E**).Meanwhile, we determined the expression of these 26 genes in human retina utilizing the BioGPS, thereby enhancing the precision and specificity of our findings Excluding CASP4, MALAT1, TAX1BP1, and ULK3, the expression levels of the remaining 22 genes in the retina surpassed the average levels observed across various systemic tissues and organ systems, underscoring their distinct and potentially significant roles within retinal physiology. Among the identified genes, CALCOCO2, CTSB, CXCR4, FYCO1, and IKBKB displayed expression levels in human retina exceeding three-fold the median, indicative of a notable enrichment of these autophagy-related genes within retinal tissue, suggesting their pivotal roles in retinal homeostasis and potentially disease processes (**Table 2**).

**Table 2:** Expression levels of dierentially expressed autophagy-related genes identified by BioGPS in retinal tissues.

Gene	Expression level^a^	Median^b^	Gene	Expression level^a^	Median^b^
>3×M					
*CALCOCO2*	285.40 ± 86.95	69.9	*CTSB*	884.62 ± 332.09	46.8
*CXCR4*	16.83 ± 2.88	3.3	*FYCO1*	108.55 ± 40.13	16.3
*IKBKB*	68.75 ± 7.47	17.7			
>1×M					
*ARNT*	3.98 ± 0.310	3.2	*ATF4*	934.30 ± 64.650	848.1
*CASP1*	10.28 ± 0.360	9.1	*DNAJB1*	171.45 ± 4.630	116.1
*FAS*	6.43 ± 0.285	5.7	*HDAC6*	4.30 ± 0.200	3.5
*HSP90AA1*	2084.88 ± 686.59	1829.7	*MAP2K7*	8.53 ± 0.685	7.1
*MAPK8*	6.22 ± 0.390	5.23	*MAPK8IP1*	4.60 ± 0.250	3.8
*MAPT*	11.82 ± 1.49	5.6	*PFN1*	576.33 ± 154.28	257.5
*STK11*	2.20 ± 0.150	1.8	*TARDBP*	88.92 ± 16.34	60.9
*TECPR2*	17.90 ± 0.950	6.3	*TSC1*	174.60 ± 24.85	92.5
*TSC2*	4.62 ± 0.340	3.8			
<1×M					
*CASP4*	10.83 ± 0.635	11.3	*MALAT1*	226.57 ± 57.63	246
*TAX1BP1*	181.93 ± 20.43	191.1	*ULK3*	5.88 ± 0.090	6.1

^a^The “Expression level” represents the expression level of ARGs in human retinal tissue; ^b^The “median” represents the average expression level of ARGs in all tissues of human and can be used as a reference for assessing retina-specific enrichment in humans. All data above from BioGPS.

### 3.3 GO and KEGG pathway enrichment analyses

**Figure 3:**
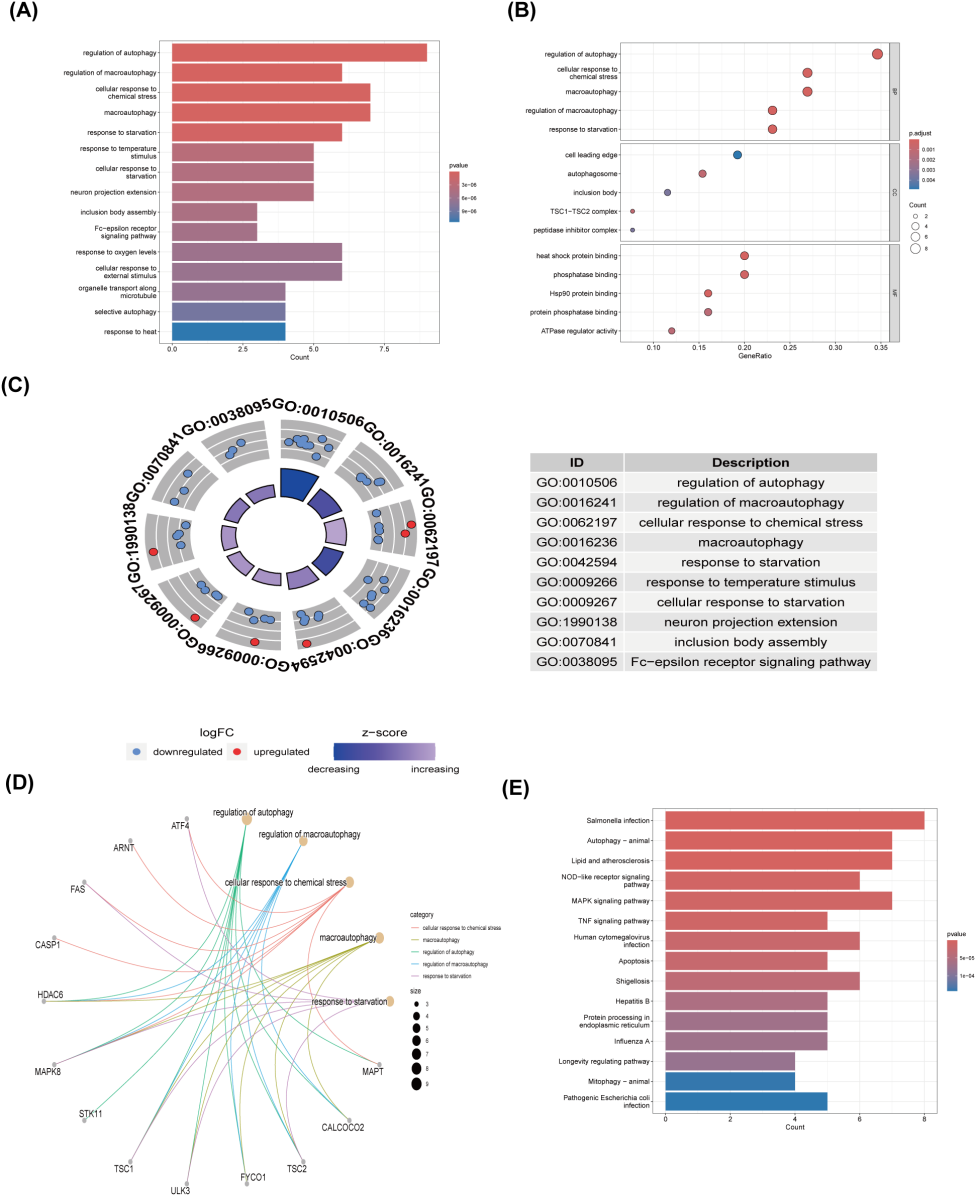
GO and KEGG enrichment analysis of 26 differentially expressed autophagy-related genes. **(A)** Bar plot of enriched GO terms. **(B)** Bubble plot of enriched GO terms. **(C)** Eight Diagrams of enriched GO terms. **(D)** Common genes in the most top enriched pathways. **(E)** Bar plot of enriched KEGG terms. GO, Gene Ontology; BPs, biological processes; CCs, cellular components; MFs, molecular function. KEGG, Kyoto Encyclopedia of Genes and Genomes.

Employing the GO and KEGG databases, we conducted a comprehensive functional enrichment analysis of 26 ARGs, providing insights into their biological roles and pathways in DR. The results of GO enrichment analysis indicated that the differential genes were significantly enriched in 404 biological processes (BP), 67 cellular components (CC), and 69 molecular functions (MF). Among the most prominent terms identified, we observed significant enrichment in: BP such as autophagy regulation, cellular response to chemical stress, macroautophagy, macroautophagy regulation, and starvation response; CC including cell leading edge, autophagosome, and inclusion body; and MF encompassing heat shock protein binding, phosphatase binding, Hsp90 protein binding, protein phosphatase binding, and ATPase regulatory activity (**Figures 3A–C**). These are all closely related to the occurrence of autophagy, indicating that autophagy plays an important role in the development of DR. Within the most prominently enriched pathways, we identified a cohort of 13 genes that play pivotal roles: ATF4, ARNT, FAS, CASP1, HDAC6, MAPK8, STK11, TSC1, ULK3, FYCO1, TSC2, CALCOCO2, and MAPT (**Figures 3D**). These genes are important in regulating key pathways and provide us with deeper insights. Furthermore, the KEGG analysis illuminated a notable enrichment of ARGs primarily within the context of Salmonella infection, autophagy in animals, lipid metabolism and atherosclerosis, Nod-like receptor signaling cascades, as well as MAPK signaling pathways (**Figures 3E**). This description not only reaffirms the critical role of ARGs in these biological processes, but also provides a novel perspective by emphasizing their complex interactions that may influence DR development through the process of autophagy, setting our findings apart from previous studies.

### 3.4 Constructing a PPI network and identifying hub genes

**Figure 4:**
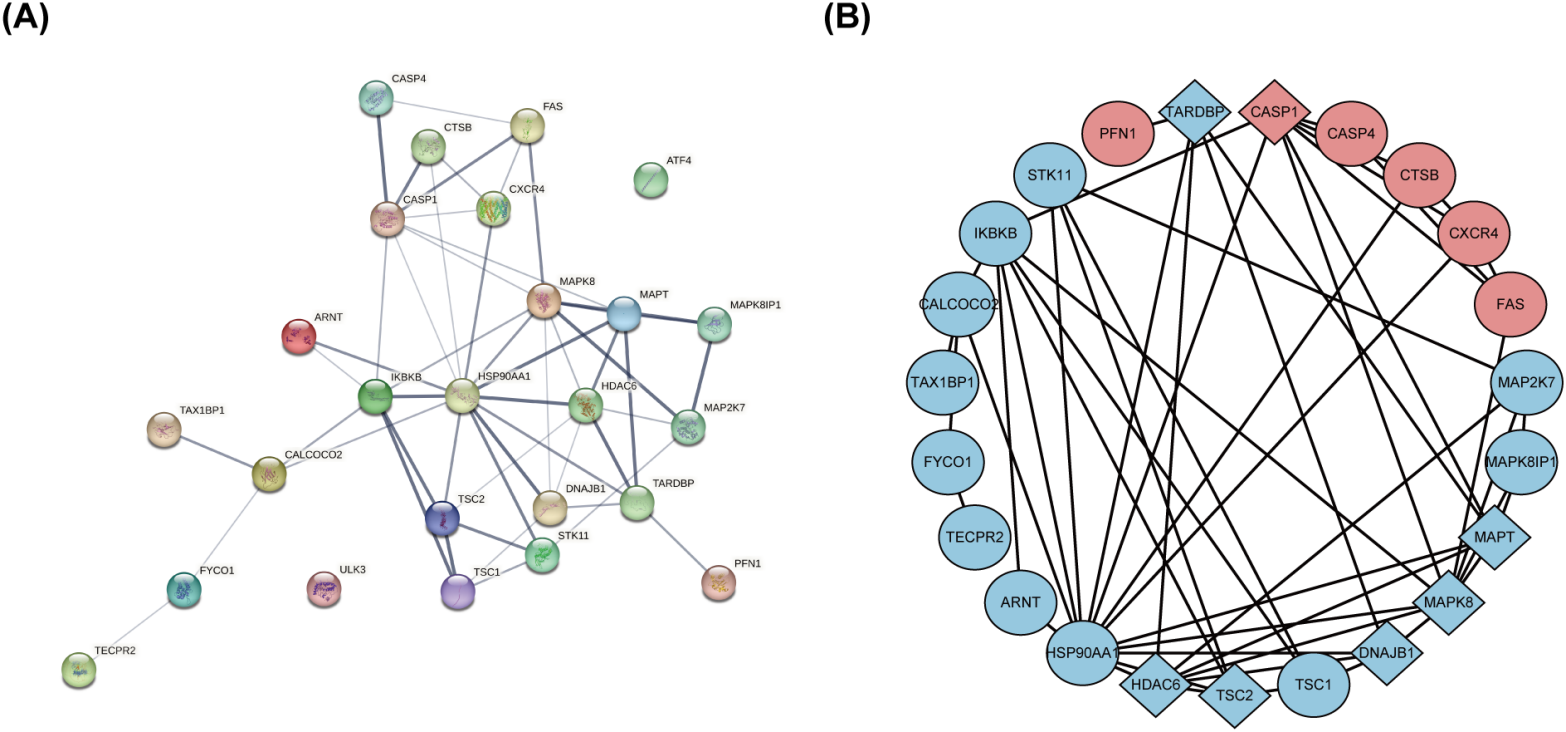
The protein-protein interaction network of 26 differentially expressed autophagy-related genes. **(A)** The PPI network of 26 differentially expressed ARGs was constructed by using String database. It contains 25 nodes and 52 edges. The average node degree is 4.16, and the PPI enrichment P-value is less than 3.68e-12. **(B)** The PPI network processed with CytoScape software consists of 26 ARGs. Blue represents downregulated genes, and red represents upregulated genes. Rhombic shape represents the hub genes; ellipse represents others. PPI,protein-protein interaction; ARGs, differentially expressed autophagy-related genes.

**Figure 5:**
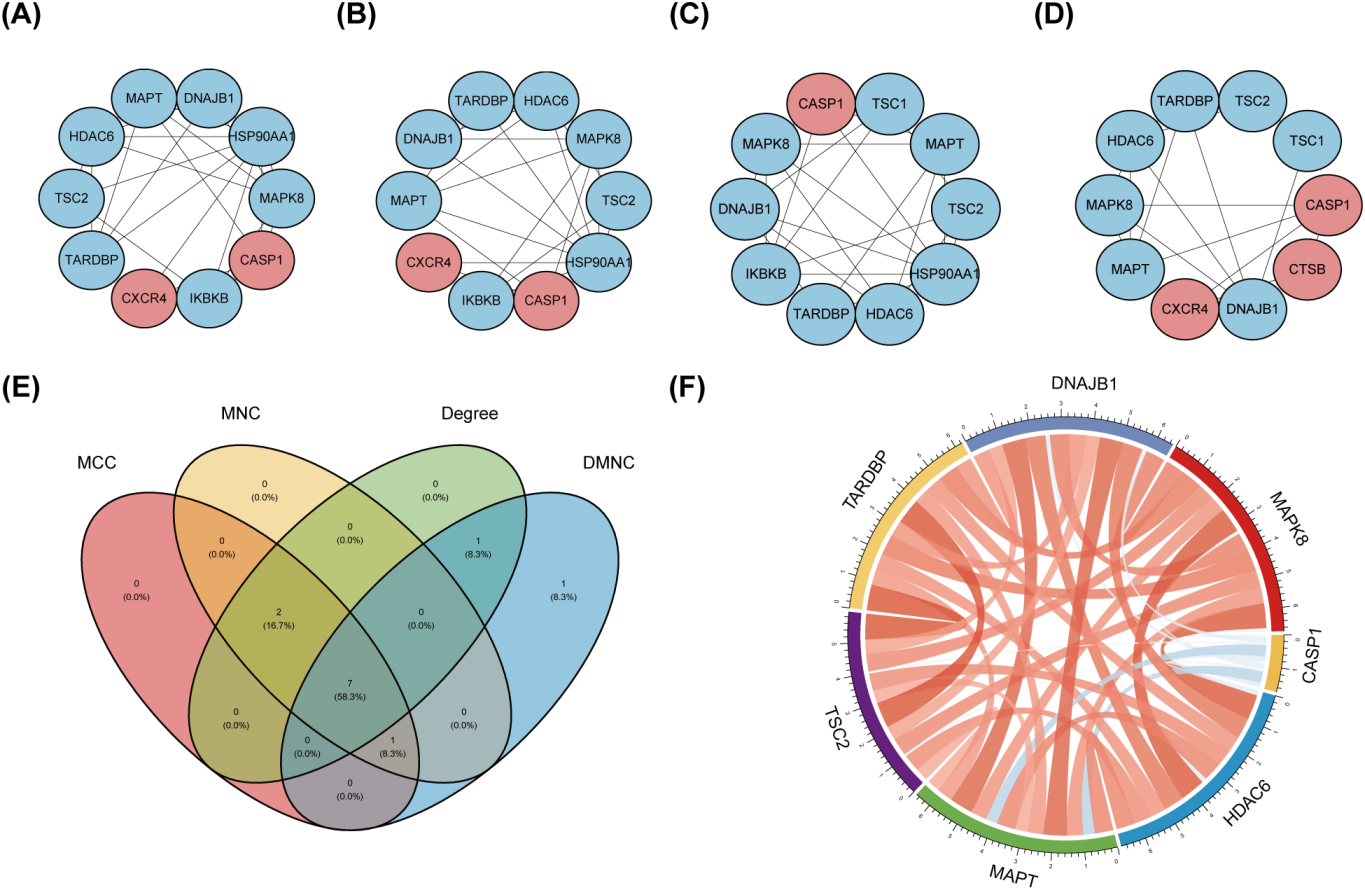
Identification and correlation analysis of hub genes. **(A-D)** The top 10 hub genes were identified through the protein-protein interaction network map using four algorithms: maximal clique centrality (MCC), maximal neighborhood centrality (MNC), degree, and density-based maximal clique (DMNC). **(E)** Venn shows 7 autophagy-related hub genes (ARHGs) obtained by four algorithms. **(F)** Chord diagram of 7 ARHGs. Red represents positive correlation, blue represents negative correlation, and the darker the color or the thicker the line, the higher the correlation intensity.MCC, maximal clique centrality; MNC, maximal neighborhood centrality; DMNC, density-based maximal clique; ARHGs, autophagy-related hub genes.

The PPI network, constructed via the intricate interplay among individual proteins, holds paramount significance in elucidating the multifaceted functions and interconnectivity of proteins, thereby advancing our comprehension of biological processes and mechanisms (22, 31). To gain a deeper insight into the intricate interplay among ARGs, we harnessed the STRING platform to curate a PPI network, integrating these genes into a comprehensive framework for analysis (**Figure 4A**). Subsequently, we derived a PPI network comprising 25 distinct nodes interconnected by 52 edges, yielding an average node degree of 4.16, indicative of a moderately dense and intricately woven interaction landscape. Among the 26 genes under investigation, one solitary gene stands apart, exhibiting no discernible associations with other gene and thus failing to contribute to the formation of a molecular network. (**Figure 4B**). To delve deeper into our analysis, we leveraged the cytoHubba plug-in within the CytoScape software, harnessing its advanced capabilities for a more profound exploration of the data.These ARGs were screened according to MCC mode, MNC mode, Degree and DMNC mode, and the top ten hub genes were selected (**Figures 5A–D**, **Table 3**). The genes that were consistently identified by the intersection of the four employed algorithms were selected as the ARHGs, thereby ensuring a rigorous and specialized approach to gene selection. We obtained 7 ARHGs, among which MAPK8, HDAC6, MAPT, TSC2, DNAJB1 and TARDBP are down-regulated genes,CASP1 is an up-regulated gene (**Figure 5E**). We postulate that alterations in these ARHGs may be intricately linked to the initiation and progression of DR, underscoring their pivotal role in the pathophysiological mechanisms. Furthermore, we conducted a correlation analysis of these 7 genes utilizing a chord diagram, which unveiled a robust interconnectivity among them, indicative of their strong associative nature (**Figure 5F**).

**Table 3:** Top ten hub genes obtained by four algorithms of Cytohubba.

MCC	MNC	Degree	DMNC
*HSP90AA1*	*HSP90AA1*	*HSP90AA1*	*DNAJB1*
*MAPK8*	*MAPK8*	*MAPK8*	*TARDBP*
*CASP1*	*CASP1*	*CASP1*	*CTSB*
*IKBKB*	*IKBKB*	*IKBKB*	*MAPT*
*HDAC6*	*HDAC6*	*HDAC6*	*CXCR4*
*MAPT*	*MAPT*	*MAPT*	*HDAC6*
*TSC2*	*TSC2*	*TSC2*	*TSC2*
*CXCR4*	*CXCR4*	*DNAJB1*	*CASP1*
*DNAJB1*	*DNAJB1*	*TARDBP*	*MAPK8*
*TARDBP*	*TARDBP*	*TSC1*	*TSC1*
*HSP90AA1*	*HSP90AA1*	*HSP90AA1*	*DNAJB1*
*MAPK8*	*MAPK8*	*MAPK8*	*TARDBP*
*CASP1*	*CASP1*	*CASP1*	*CTSB*
*IKBKB*	*IKBKB*	*IKBKB*	*MAPT*
*HDAC6*	*HDAC6*	*HDAC6*	*CXCR4*
*MAPT*	*MAPT*	*MAPT*	*HDAC6*

### 3.5 Construction of a miRNA-gene-TF interaction network

**Figure 6:**
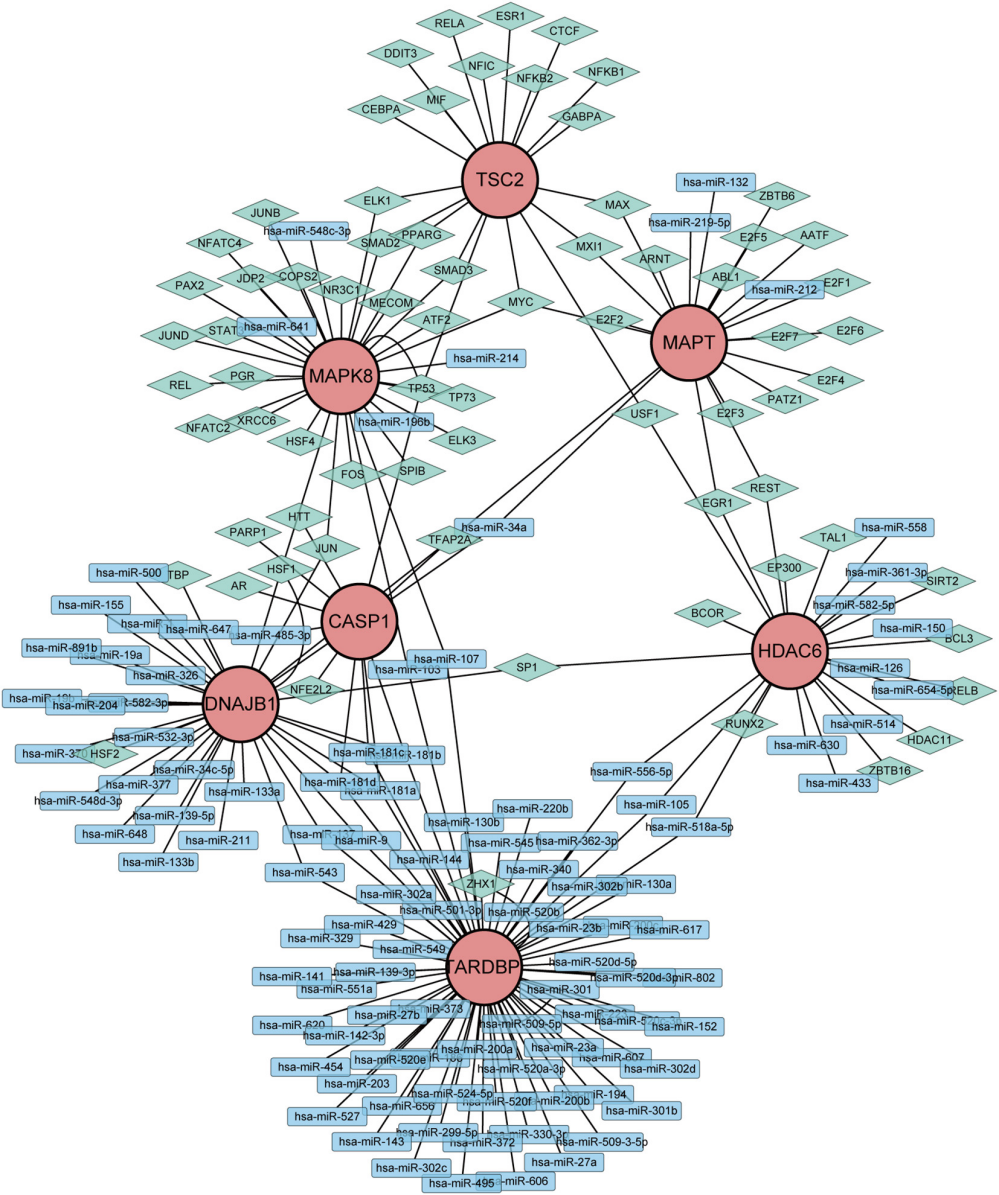
Network of miRNAs-genes-TFs interacting with hub genes. Rectangles represents miRNAs; Diamond shape represents TFs; Circles represents hub genes.

To gain further insights into the intricate interplay among genes, miRNAs, and TFs in the context of DR, we devised a comprehensive miRNA-gene-TF interaction network, thereby facilitating a deeper understanding of their dynamic regulatory landscape. Firstly, leveraging the ARHGs as a foundation, we employed the NetworkAnalyst 3.0 platform to forecast an intricate network comprising 107 miRNAs, 72 TF, and 222 interconnected edges, thus facilitating a comprehensive investigation of their regulatory relationships. Our network revealed that MAPK8 is modulated by 6 miRNAs, CASP1 by 5 miRNAs, HDAC6 by 12 miRNAs, MAPT by 4 miRNAs,DNAJB1 by 28 miRNAs, and TARDBP being regulated by a single miRNA. No direct miRNA regulation of TSC2 was observed (**Supplementary Table S3**). The regulatory landscape of these genes reveals intricate transcriptional control, with MAPK8 governed by 28 TFs, CASP1 by 6 TFs, HDAC6 by 13 TFs, MAPT by a notable 18 TFs, TSC2 by 19 TFs, DNAJB1 by 7 TFs, and TARDBP displaying an extensive regulatory network of 70 TFs. Ultimately, we visualized a novel miRNA-Gene-TF interaction network using the Cytoscape software platform, providing a comprehensive representation of the complex regulation (**Figure 6**). The common hub genes were found in this network, suggesting a profound interplay between TFs and hub genes.

### 3.6 Target drug forecasting

Leveraging the extensive resources of DGIdb, we prospectively identified potential therapeutic agents that may exert beneficial effects on DR by specifically targeting ARHGs, thereby contributing to the development of novel and targeted therapeutic strategies. With the exception of DNAJB1, our analysis founded 651 drug candidates that were associated with 6 ARHGs, showing the breadth of potential therapeutic avenues explored through this targeted approach. **Table 4** enumerates the agents that have achieved a query score of 5 or greater, or in the case of the target gene, the highest query score.

**Table 4:** Potential therapeutic agents targeting ARHGs.

Drugs	Targets	Query score	Interaction score	FDA approval
TANZISERTIB	*MAPK8*	2.87	1.03	not approved
PRALNACASAN	*CASP1*	8.61	5.38	approved
VERMISTATIN	*CASP1*	8.61	5.38	not approved
BELNACASAN	*CASP1*	8.61	5.38	not approved
CHEMBL415893	*CASP1*	8.61	5.38	not approved
BENZOHYDROXAMATE	*HDAC6*	12.91	4.76	not approved
VALPROPYLHYDROXAMICACID	*HDAC6*	8.61	3.17	not approved
CHEMBL569419	*HDAC6*	8.61	3.17	not approved
NICOXAMAT	*HDAC6*	8.61	3.17	not approved
RICOLINOSTAT	*HDAC6*	6.46	2.38	not approved
CHEMBL2181040	*MAPT*	8.61	0.28	not approved
CHEMBL2181046	*MAPT*	8.61	0.28	not approved
CHEMBL2181039	*MAPT*	8.61	0.28	not approved
CHEMBL2181041	*MAPT*	8.61	0.28	not approved
EVEROLIMUS	*TSC2*	0.44	6.34	approved
ESFLURBIPROFEN	*TARDBP*	4.3	0.55	not approved

### 3.7 Verification

**Figure 7:**
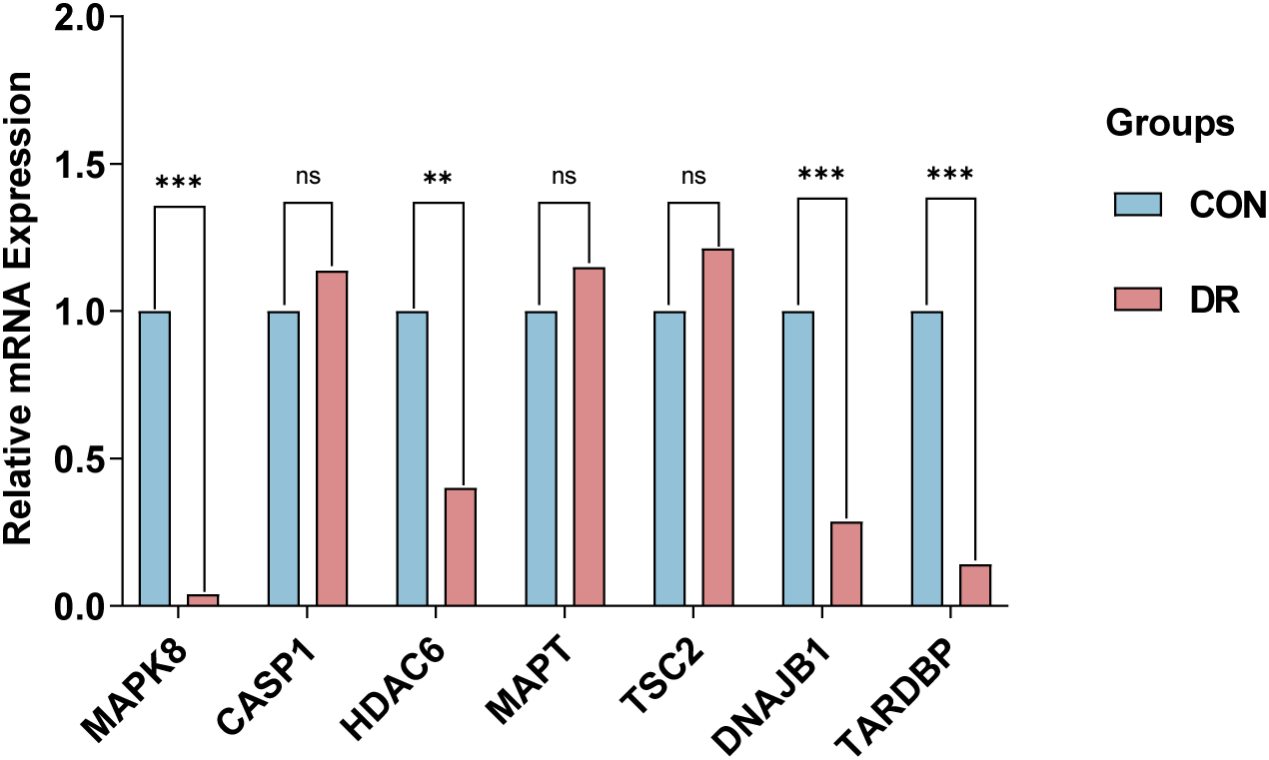
qRT-PCR experiment to verify the expression of 7 autophagy related hub genes of interest in the in ARPE-19 cells. P-values were calculated using Student’s t-test. **P < 0.01; ***P < 0.001; ns, non-significant. Blue bars represent the control group, denoted by “CON”, and red bars represent the DR group, denoted by “DR”. qRT-PCR, quantitative real-time polymerase chain reaction; ARPE-19, retinal pigment epithelial cell line.

To corroborate the robustness of our findings derived from the GSE102485 (13) dataset, we employed qRT-PCR as a validation strategy, ensuring the integrity and accuracy of our analytical outcomes. Firstly, ARPE-19 cells were cultivated under two distinct conditions: a hyperglycemic milieu comprising 25 mmol/L of anhydrous glucose and a normoglycemic setting. The ARPE-19 cells exposed to elevated glucose concentrations were employed as an *in vitro* model of DR. Conversely, ARPE-19 cells maintained in normal glucose media served as the control group, providing a baseline for comparison and elucidating the specific effects of high glucose exposure. Then, we conducted a detailed quantification of the expression of the 7 ARHGs through the utilization of qRT-PCR. Ultimately, resemble to the mRNA microarray findings reported in the GSE102485 dataset, our study observed a pronounced downregulation of mRNA expression levels for MAPK8, HDAC6, DNAJB1, and TARDBP in ARPE-19 cells subjected to high glucose treatment. This result emphasizes the specific and consistent effects of hyperglycemic status on these genes, thus providing a new molecular basis for RPE dysfunction in DR. Intriguingly, despite predictions from our bioinformatics analysis that CASP1, MAPT, and TSC2 expression would be upregulated, these genes exhibited no significant alterations in their expression levels in ARPE-19 cells subjected to high glucose treatment, in contrast to the control cells. This demonstrates the complexity of gene regulation in DR in response to hyperglycemic stimuli and emphasizes the need for further research into the mechanisms behind the occurrence of this condition (**Figure 7**).

**Figure 8:**
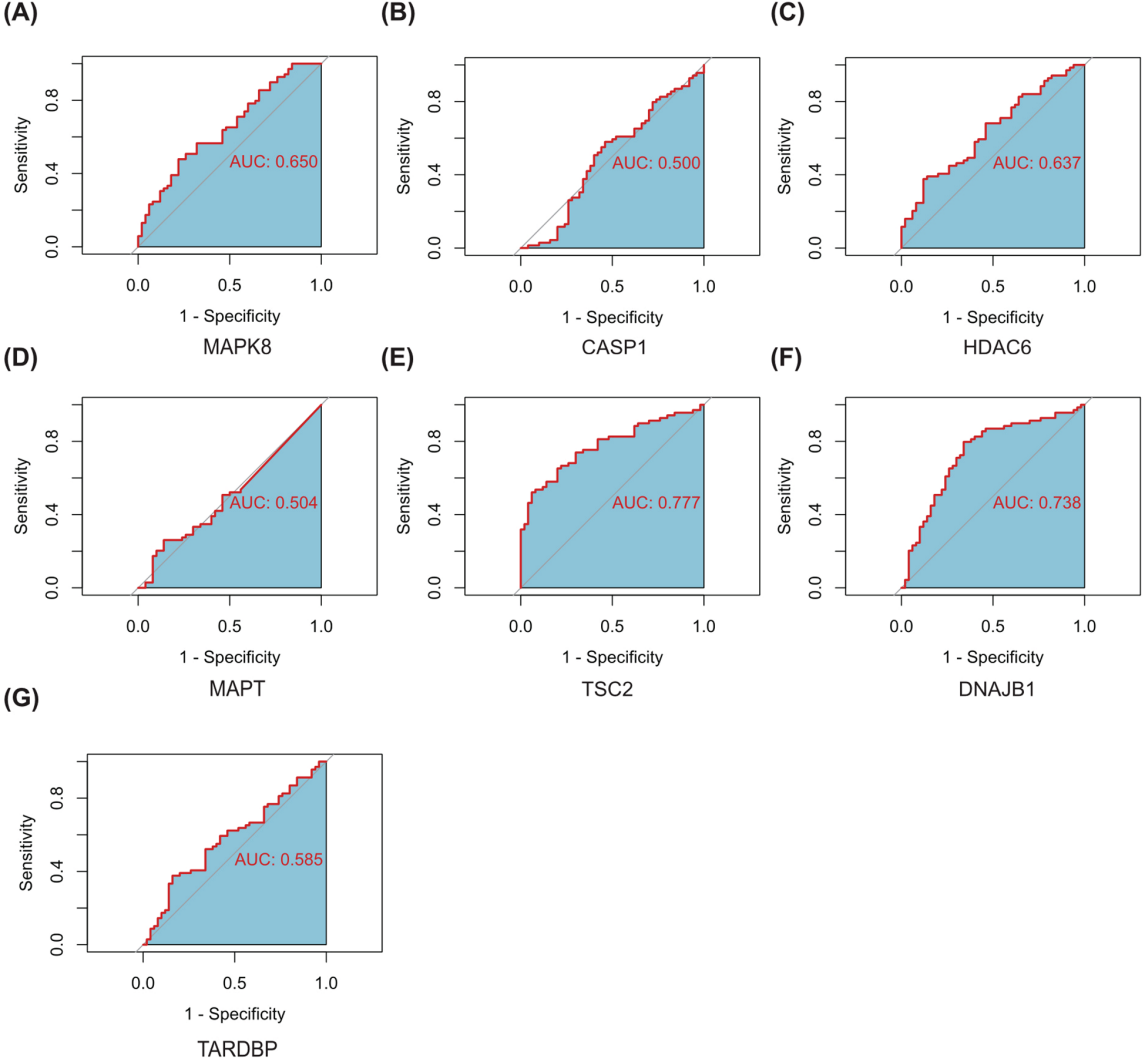
ROC curves of the 7 autophagy-related hub genes for the diagnosis when distinguishing DR from normal **(A–G)**. AUC, Area Under Curve.

Subsequently, we delved into the diagnostic potential of these seven genes in DR, constructing ROC curves to evaluate their discriminatory power and clinical utility. The analysis of ROC curves revealed that in GSE221521(peripheral blood samples), the AUROC of MAPK8, CASP1, HDAC6, MAPT, TSC2, DNAJB1 and TARDBP were 0.650, 0.500, 0.637, 0.504, 0.777, 0.783 and 0.585 respectively, when differentiating patients with DR from controls (**Figure 8**).Our finding indicate that MAPK8, HDAC6, TSC2, and DNAJB1 have some diagnostic benefits as new biomarkers of DR, suggesting their potential as invaluable tools for early detection and prognosis assessment.

## 4 Discussion

Diabetes has long been recognized as a pervasive public health challenge on a global scale. DR emerging as a distinct microvascular complication that poses a significant threat to vision, accounting for a substantial proportion of blindness cases in numerous countries. This highlights the necessity to address DR as a significant public health concern that requires innovative strategies for prevention, early detection, and effective management (2, 32). The etiology of DR is multifaceted, with the prevailing paradigm positing DR as a neurovascular disorder of the retina, characterized primarily by microvascular alterations (1). In its early stages, DR presents with a myriad of pathological transformations, encompassing retinal neurodegeneration, endothelial dysfunction, and notably, angiogenesis, which serves as a hallmark of DR, subsequently progressing to fibrovascular proliferation and hemorrhage. Prolonged hyperglycemia acts as a pivotal driver, fostering the thickening of retinal microvascular basement membranes, endothelial cell depletion, heightened vascular permeability, and the induction of neovascularization (33). This intricate interaction indicates the necessity of a nuanced understanding of the pathogenesis of DR in order to facilitate the development of targeted therapeutic interventions. Recent investigations have illuminated a pivotal role for autophagy in the initiation and progression of DR (7). Despite the intricate molecular mechanisms underlying DR remaining largely unravelled, a growing body of evidence underscores the intricate interplay between autophagy and factors such as oxidative stress, hypoxia, endoplasmic reticulum stress, and other diabetes-related perturbations, all of which are implicated in the pathogenesis of DR (34). The intersection of these pathways highlights the potential of modulating autophagy as a novel therapeutic pathway in the treatment of DR and suggests the need to further explore the role of autophagy in DR.

Autophagy is a fundamental cellular mechanism that maintains the stability of the intracellular environment and external homeostasis by selectively degrading intracellular components. This complex process is coordinated by a series of signaling cascades, most notably the activation of mammalian target of rapamycin (mTOR) and AMP-activated protein kinase (AMPK) signaling pathways (35). Autophagy occurs in most retinal cells in patients with DR. However, autophagy is a double-edged sword: while moderate autophagy is a protective mechanism that strengthens retinal cells against the deleterious effects of reactive oxygen species (ROS), inflammatory cascades, and traumatic injury, excessive enhancement of autophagy triggers aberrant cell death, and a delicate balance must be maintained in this complex process (34). Some scholars believe that tumor necrosis factor-α (TNF-α) released by Müller cells activates the epidermal growth factor receptor (EGFR)/p38 cytokine-activated protein kinase (MAPK)/nuclear factor-κB (NF-κB)/p62 pathway, which increases mitochondrial autophagy and apoptosis in retinal pigment epithelial (RPE) cells under high glucose conditions. This damage to the RPE accelerates the development of DR, suggesting that a complex interaction between autophagy and DR is critical for the development of DR (36). In addition, Liu et al. found an interesting observation that the expression of glial cell maturation factor-β (GMFB) was significantly up-regulated in the vitreous humor of patients diagnosed with early DR. Notably, as a stress-responsive protein, extracellular GMFB plays a key role in regulating autophagy and iron homeostasis, which ultimately affects the important physiological functions of RPE cells. The role of autophagy activation in RPE cells highlights its potential to accelerate the pathological progression of DR, providing new insights into the complex mechanisms of this disease (37). In conclusion, the role of autophagy in various types of retinal cells during the development of DR highlights the complexity of the underlying mechanism, which remains enigmatic. In order to reveal the exact role of autophagy in the pathogenesis of DR, more in-depth explorations are necessary to gain a more nuanced understanding of the dynamic interactions between the autophagic pathway and the DR environment.

In our study, we identified 26 ARGs by analyzing the DR dataset and autophagy-related genes using a bioinformatics approach. Subsequently, we performed correlation analyses of these 26 ARGs, and the results revealed strong correlations among them. We used BioGPS to determine the expression of the above 26 genes in the human retina. The analysis showed that CALCOCO2, CTSB, CXCR4, FYCO1 and IKBKB were significantly enriched in retinal tissue. This finding highlights the potential association of these genes with retinal function and homeostasis. The results of GO and KEGG enrichment analyses emphasize the profound association of these genes with important biological processes, including in particular the regulation of autophagy, regulation of macroautophagy, and macroautophagy. This finding emphasizes the central position of these genes in the autophagy network. In addition, KEGG pathway analysis revealed the involvement of these ARGs genes in the Nod-like receptor signaling pathway and MAPK signaling pathway. Evidence from DR studies suggests that these pathways have critical functions, in perfect agreement with our predictive analysis (38–40). Autophagy is a regulator of inflammasome activation through downregulation of NF-κB and related signaling cascades. A seminal study by Piippo N, et al. demonstrated that autophagy inhibition in RPE cells promotes extracellular release of NOD-like receptor protein 3 (NLRP3) and subsequent inflammasome activation, thereby clarifying autophagy’s central role in modulating ocular inflammatory responses (41, 42). Subsequently, 7 ARHGs with DR were identified, including MAPK8, CASP1, HDAC6, MAPT, TSC2, DNAJB1, and TARDBP. After comprehensive analysis using the PPI network and cytoHubba, we identified seven autophagy-related hub genes (ARHGs) that are significantly associated with DR. These genes, namely MAPK8, CASP1, HDAC6, MAPT, TSC2, DNAJB1, and TARDBP, represent a unique set of biomarkers that may hold the key to unraveling the involvement of autophagy in the pathogenesis of DR.MAPK8 has been demonstrated to activate the FoxO signalling pathway, which plays a pivotal role in the pathogenesis of DR by regulating antioxidant enzymes to maintain cellular redox homeostasis (43). Yu et al. unveiled a significant upregulation of the lncRNA myocardial infarction-associated transcript (MIAT) in human pericytes under DR conditions. This upregulation promoted heat-induced apoptosis in pericytes under DR conditions through a mechanism involving the regulation of miR-342-3p, a microRNA targeting CASP1. This complex regulatory cascade underscores the contributory role of CASP1 in the pathogenesis of DR (44). Furthermore, a series of studies demonstrated that the upregulation of CASP1 in an *in vitro* model of DR is intricately intertwined with the signaling cascade of vascular endothelial growth factor receptor 1 (45, 46). Additionally, evidence indicates that HDAC6 levels are elevated in the retinas of diabetic rats relative to healthy controls, with this elevation occurring concurrently with oxidative stress-mediated damage-induced autophagy. GLP-1 treatment attenuated oxidative stress-induced autophagy in DR via the GLP-1R-ERK1/2-HDAC6 signaling pathway, suggesting that HDAC6 plays a role in DR (47). Yi et al. showed that depletion of the TARDBP gene-encoded TAR DNA binding protein 43(TDP-43) in the DR protects RGC-5 cells from oxidative stress-mediated apoptosis and autophagy through inhibition of its target HDAC6 (48). Next, we constructed a miRNAs-Genes-TFs interaction network of seven hub genes. Finally, we utilized HG-treated ARPE-19 cells as an *in vitro* model of DR to ascertain whether hub genes exhibited differential expression. The expression of four key genes, namely MAPK8, HDAC6, DNAJB1, and TARDBP, were found to be differentially expressed in DR samples compared to controls by RT-qPCR, thereby confirming previous bioinformatics predictions. Furthermore, MAPK8, HDAC6, DNAJB1, and TSC2 may serve as promising biomarkers for DR, exhibiting high diagnostic sensitivity and specificity, as corroborated by ROC curve analysis.

Mitogen-activated protein kinase 8 (MAPK8), colloquially referred to as c-Jun N-terminal kinase (JNK), stands as a pivotal member within the MAPK superfamily, orchestrating a broad spectrum of physiological processes such as inflammatory cascades, cellular differentiation, proliferation, and apoptosis. Aberrant regulation of MAPK8 has garnered attention as a contributing factor in a myriad of pathological conditions, encompassing diabetes mellitus, oncogenesis, autoimmune disorders, cardiac hypertrophy, and asthmatic phenotypes, thereby underscoring its pivotal role in maintaining cellular homeostasis and disease pathogenesis (49, 50). Prior research has demonstrated that *in vitro* model of diabetic rats, EPO maintains the integrity of the outer blood-retina barrier by down-regulating JNK signalling and thus up-regulating the expression of ZO-1 and occludin in RPE cells, which may be a potential therapeutic target to slow down the progression of DR (51). In a study conducted by Guma M. et al., intravitreal injection of a targeted JNK inhibitor was observed to significantly reduce retinal vascular endothelial growth factor (VEGF) expression while attenuating pathological retinal neovascularization. This study highlights the central role of JNK1 in retinal neovascularization and points to its potential as an innovative pharmacological target for the treatment of a wide range of diseases characterized by aberrant angiogenesis, including DR. As a result, therapeutic strategies for the disease are being advanced (52). The results of our study suggest that MAPK8 may be associated with autophagic processes in DR. However, further research is needed to elucidate the precise role of MAPK8 in DR.

Histone deacetylase 6 (HDAC6) is a class IIb histone deacetylase that plays a significant role in regulating the acetylation state of nuclear and cytoplasmic proteins, thereby exerting important biological functions (53). The full implications of HDAC6 on DR have yet to be fully elucidated. It is noteworthy that HDAC6, as an epigenetic regulator of histone deacetylases, is closely related to autophagy. This suggests that it plays a key role in regulating retinal homeostasis and potentially influencing the pathogenesis of DR (54). Some scholars have proposed that GLP-1 may alleviate DR by reducing autophagy via the GLP-1 R-ERK 1/2-HDAC6 signaling pathway, indicating that the HDAC 6 signaling pathway may be a crucial factor in DR (47). In a study conducted by Leyk and colleagues, HDAC6 was observed to have pro-oxidative properties in an animal model of retinal neurodegenerative disease. This finding suggests that HDAC6 may be associated with retinal diseases characterized by oxidative stress, particularly DR (55). Furthermore, there is compelling evidence that HDAC6 expression and activity are elevated in human diabetic retinas obtained from postmortem donors, as well as in an experimental model of streptozotocin (STZ)-induced DR in rats. Consequently, HDAC6 may serve as a pivotal regulator in the pathogenesis of DR. Furthermore, HDAC6 activation has been demonstrated to modulate hyperglycemia-induced oxidative stress in the retina, which can result in retinal microangiopathy and may even precipitate DR (56). Interestingly, our study indicates that based on bioinformatics analysis, HDAC6 expression is diminished in the DR group and may be implicated in the process of autophagy in DR. This finding may be the result of a limited number of samples or the balance of multiple processes involved such as autophagy. However, the specific mechanism of HDAC6 involvement in DR deserves further investigation.

The DNAJ heat shock protein family member B1 (DNAJB1), an integral constituent of the 40-kDa heat shock protein cohort, has emerged as a multifaceted regulator implicated in diverse cellular machineries. Its functions include pivotal roles in the proteasome-mediated protein degradation pathway, coordination of the endoplasmic reticulum stress response, and, more intriguingly, recent findings have associated DNAJB1 with the progression of neoplastic disorders, emphasizing its significance as a versatile modulator of cellular homeostasis and disease pathogenesis (57–59). It has been demonstrated that elevated levels of DNAJB1 are linked to insulin resistance (60). Additionally, DNAJB1 levels are reduced in response to euglycemia, indicating that heat shock proteins are responsive to basal stress. Currently, no studies of DNAJB1 in DR have been identified. Further investigation is required to elucidate the specific mechanism of DNAJB1 involvement in DR.

It is of the utmost importance to acknowledge the inherent limitations of this study. To begin with, the sample size procured from public databases for gene expression profiling is somewhat constrained, which may introduce biases from individual variability that could compromise the broad applicability of our analytical findings. In addition, while our study identified MAPK8 and HDAC6 as autophagy-related biomarkers in DR, several limitations should be acknowledged. First, our analyses utilized retinal tissue datasets and an *in vitro* model lacking clinical staging data, which precludes determination of their phase-specific predictive utility (e.g., non-proliferative vs. proliferative DR). Second, while the ARPE-19 high-glucose model recapitulates key DR features, it does not fully capture the dynamic progression of human DR across distinct clinical stages. To address these gaps, future studies should incorporate longitudinal cohorts with stratified DR severity to delineate stage-dependent biomarker roles. Additionally, single-cell RNA sequencing of staged human retinal tissues could resolve autophagy heterogeneity among cellular subpopulations during DR progression. Finally, their specificity relative to other retinopathies (e.g., AMD) remains to be investigated. Future studies comparing multi-disease cohorts and single-cell sequencing of retinal cell subtypes will help disentangle shared versus DR-specific autophagy mechanisms.

## 5 Conclusions

The present study commenced with a preliminary identification of pivotal autophagy-associated genes, namely MAPK8, HDAC6, and DNAJB1, in the context of DRthrough rigorous bioinformatic analyses. This initial screen was subsequently validated in an *in vitro* model of diabetes utilizing qRT-PCR, thereby offering a novel perspective into the intricate molecular mechanisms governing autophagy during DR pathogenesis. Nevertheless, the precise contributions of these autophagy-related genes to DR remain unclear and require further investigation through comprehensive *in vivo* and *in vitro* experimentation. This will advance our understanding of this complex disease process.

## Data Availability

Publicly available datasets were analyzed in this study. This data can be found here: http://www.ncbi.nlm.nih.gov/geo/.
