# Supplementary material for: MAPK8 and HDAC6: potential biomarkers related to autophagy in diabetic retinopathy based on bioinformatics analysis
Source: Front Endocrinol (Lausanne). 2025 May 21;16:1487007. doi: 10.3389/fendo.2025.1487007 (PMC12133481; doi:10.3389/fendo.2025.1487007)
Supplement: Supplementary file 1 [file DataSheet1.zip › TableS1-3/TableS1.docx]

**Supplementary Table S1 | Primers used in the study for quantitative PCR.**

| **Gene** | **Primer Sequence (from 5' to 3')** |
| --- | --- |
| *MAPK8* | Forword: 5'-ACTACAGAGCACCCGAGGTCATC-3'  Reverse: 5'-TTTCTCCCATGATGCACCCAACTG-3' |
| *CASP1* | Forword: 5'-CACACCGCCCAGAGCACAAG-3'  Reverse: 5'-TCCCACAAATGCCTTCCCGAATAC-3' |
| *HDAC6* | Forword: 5'-GGCAGCGAAGAAGTAGGCAGAAC-3'  Reverse: 5'-AGATTGGGGATAGAGCGGGGAAC-3' |
| *MAPT* | Forword: 5'- AGAACGCCAAAGCCAAGACAGAC-3'  Reverse: 5'-CATTGCTGAGATGCCGTGGAGAC-3' |
| *TSC2* | Forword: 5'- TGTCCGAACGAGGTGGTGTCC-3'  Reverse: 5'-AGGTCTGGAGCTGCTGAAGGAG-3' |
| *DNAJB1* | Forword: 5'- GGGAGGAAGGCATGGACATTGATG-3'  Reverse: 5'- CTTCTTTCGGGCGGGCTCTTG-3' |
| *TARDBP* | Forword: 5'-GGGTGGTGGGATGAACTTTGGTG-3'  Reverse: 5'-CTGGTTCTGCTGGCTGGCTAAC-3' |
| *β-Actin* | Forword: 5'-GGCATGGGTCAGAAGGATT-3'  Reverse: 5'-TGGTGCCAGATTTTCTCCA-3' |
