# Supplementary material for: MAPK8 and HDAC6: potential biomarkers related to autophagy in diabetic retinopathy based on bioinformatics analysis
Source: Front Endocrinol (Lausanne). 2025 May 21;16:1487007. doi: 10.3389/fendo.2025.1487007 (PMC12133481; doi:10.3389/fendo.2025.1487007)
Supplement: Supplementary file 1 [file DataSheet1.zip › TableS1-3/TableS2.docx]

**Table S2 | 344 autophagy-related genes**

| **Gene Symbol** | | | | | | |
| --- | --- | --- | --- | --- | --- | --- |
| VMA21 | TP53 | LRRK2 | IFNG | DAPK3 | DAPK2 | MBTPS2 |
| ATG5 | WIPI1 | GSK3B | GAA | TRAPPC4 | DDIT3 | NAF1 |
| BECN1 | VCP | DAPK1 | STK11 | ATP13A2 | DIRAS3 | NAMPT |
| ATG16L1 | PRKAA1 | TP53INP1 | PARP1 | RAB39B | DLC1 | NCKAP1 |
| ULK1 | MIR7-3HG | TMX2-CTNND1 | RPS6KB1 | RIPK1 | DNAJB1 | NKX2-3 |
| AMBRA1 | ATG12P1 | PARK7 | HTT | MAP1LC3B2 | DNAJB9 | NLRC4 |
| ATG12 | ELAPOR2 | SNCA | BAG3 | TRIM65 | EDEM1 | NPC1 |
| ATG7 | PIK3R4 | TAX1BP1 | NOD1 | BNIP3L | EEF2 | NRG1 |
| ATG4B | WIPI2 | CISD2 | TLR4 | ATM | EEF2K | NRG2 |
| SQSTM1 | C9orf72 | TMEM59 | CASP8 | NUPR1 | EIF2AK2 | NRG3 |
| ATG14 | OPTN | NRBF2 | DEPTOR | RPS27A | EIF2AK3 | P4HB |
| ATG13 | VMP1 | MALAT1 | SIRT2 | XBP1 | EIF2S1 | PARK2 |
| ATG3 | ATG3P1 | WDR41 | TRIM21 | IGF1 | EIF4EBP1 | PEA15 |
| ATG9A | HDAC6 | TARDBP | MCL1 | VDAC1 | EIF4G1 | PELP1 |
| ATG4A | TBK1 | TUFM | RAB8A | APOL1 | ERBB2 | PEX14 |
| ATG4D | ATG12P2 | HIF1A | IL6 | ARNT | ERN1 | PEX3 |
| ATG4C | AKT1 | TRAF6 | PVT1 | ARSA | ERO1L | PPP1R15A |
| ATG10 | CALCOCO2 | MFN2 | KRAS | ARSB | FADD | PRKAR1A |
| ATG9B | SIRT1 | MEG3 | EGFR | ATF4 | FAM48A | PRKCD |
| MAP1LC3B | ATG4AP1 | CASP3 | PFN1 | ATF6 | FAS | PRKCQ |
| EPG5 | PRKN | LAMP1 | TRC-GCA24-1 | ATIC | FKBP1A | PTK6 |
| ATG2B | MTCL2 | MLST8 | HULC | BAG1 | FKBP1B | RAB11A |
| ULK2 | SCARNA5 | RAB7A | EVA1A | BAK1 | FOS | RAB1A |
| DRAM1 | NFE2L2 | BCL2L1 | VAMP8 | BID | GAPDH | RAB24 |
| MTOR | HSPA8 | EMSLR | WDR45B | BIRC5 | GNAI3 | RAB33B |
| MAP1LC3A | PRKAA2 | MAPK8 | ENDOG | BIRC6 | GNB2L1 | RAC1 |
| DRAM2 | SOD2-OT1 | TECPR1 | HDAC1 | BNIP1 | GOPC | RAF1 |
| NBR1 | LOC727709 | STING1 | USP13 | C12orf44 | GRID1 | RB1 |
| ATG16L2 | UBQLN2 | FUS | CTSB | C17orf88 | GRID2 | RELA |
| ATG101 | WDFY3 | PTEN | TRIM27 | CAMKK2 | HGS | RGS19 |
| ATG2A | H19 | MAPK14 | MAPT | CANX | HSP90AB1 | RHEB |
| LAMP2 | SH3GLB1 | TGFB1 | FOXK1 | CAPN1 | HSPB8 | SAR1A |
| RUBCN | TECPR2 | ZKSCAN3 | SRC | CAPN10 | IKBKB | SERPINA1 |
| PIK3C3 | PINK1 | MAPK1 | PRKAB1 | CAPN2 | IKBKE | SESN2 |
| RUBCNL | FOXO3 | SMCR8 | MYC | CAPNS1 | IL24 | SPHK1 |
| BCL2 | LINC01672 | ULK3 | RAB5A | CASP1 | ITGA3 | SPNS1 |
| MAP1LC3C | WDR45 | USP10 | XIST | CASP4 | ITGA6 | ST13 |
| GABARAPL1 | KEAP1 | UBQLN4 | BAX | CCL2 | ITGB1 | TM9SF1 |
| RB1CC1 | RPTOR | STX17 | HSPA5 | CCR2 | ITGB4 | TMEM49 |
| GABARAP | BNIP3 | NFKB1 | TNF | CD46 | ITPR1 | TMEM74 |
| DEPP1 | CERNA3 | TSC2 | FOXO1 | CDKN1A | KIAA0226 | TNFSF10 |
| ELAPOR1 | BDNF-AS | GAS5 | TRIM5 | CDKN1B | KIAA0652 | TP63 |
| EI24 | NOD2 | HDAC10 | HMOX1 | CDKN2A | KIAA0831 | TP73 |
| HMGB1 | STAT3 | CTSD | DNM1L | CFLAR | KIF5B | TSC1 |
| FYCO1 | BECN2 | NLRP3 | DAP | CHMP4B | KLHL24 | TUSC1 |
| GABARAPL2 | UBC | CHMP2B | FLCN | CLN3 | MAP2K7 | VAMP3 |
| TFEB | UBQLN1 | MTMR14 | HNRNPA1 | CTSL1 | MAPK3 | VAMP7 |
| IRGM | TP53INP2 | SOD1 | IL1B | CX3CL1 | MAPK8IP1 | VEGFA |
| UVRAG | HSP90AA1 | TBC1D5 | PIM2 | CXCR4 | MAPK9 | WDR45L |
| ZFYVE1 |  |  |  |  |  |  |
