# Supplementary material for: MAPK8 and HDAC6: potential biomarkers related to autophagy in diabetic retinopathy based on bioinformatics analysis
Source: Front Endocrinol (Lausanne). 2025 May 21;16:1487007. doi: 10.3389/fendo.2025.1487007 (PMC12133481; doi:10.3389/fendo.2025.1487007)
Supplement: Supplementary file 1 [file DataSheet1.zip › TableS1-3/TableS3.docx]

**Table S3 | Key miRNAs that regulate hub genes predicted by NetworkAnalyst 3.0 platform.**

| **MAPK8** | **CASP1** | **HDAC6** | **MAPT** | **TSC2** | **DNAJB1** | **TARDBP** |
| --- | --- | --- | --- | --- | --- | --- |
| hsa-miR-103 | hsa-miR-181a | hsa-miR-105 | hsa-miR-132 | *NA* | hsa-miR-1 | hsa-miR-103 |
| hsa-miR-107 | hsa-miR-181b | hsa-miR-126 | hsa-miR-212 |  | hsa-miR-133a | hsa-miR-105 |
| hsa-miR-196b | hsa-miR-181c | hsa-miR-150 | hsa-miR-219-5p |  | hsa-miR-133b | hsa-miR-107 |
| hsa-miR-214 | hsa-miR-181d | hsa-miR-361-3p | hsa-miR-34a |  | hsa-miR-137 | hsa-miR-130a |
| hsa-miR-548c-3p | hsa-miR-485-3p | hsa-miR-433 |  |  | hsa-miR-139-5p | hsa-miR-130b |
| hsa-miR-641 |  | hsa-miR-514 |  |  | hsa-miR-155 | hsa-miR-137 |
|  |  | hsa-miR-518a-5p |  |  | hsa-miR-181a | hsa-miR-139-3p |
|  |  | hsa-miR-556-5p |  |  | hsa-miR-181b | hsa-miR-141 |
|  |  | hsa-miR-558 |  |  | hsa-miR-181c | hsa-miR-142-3p |
|  |  | hsa-miR-582-5p |  |  | hsa-miR-181d | hsa-miR-143 |
|  |  | hsa-miR-630 |  |  | hsa-miR-19a | hsa-miR-144 |
|  |  | hsa-miR-654-5p |  |  | hsa-miR-19b | hsa-miR-152 |
|  |  |  |  |  | hsa-miR-204 | hsa-miR-181a |
|  |  |  |  |  | hsa-miR-211 | hsa-miR-181b |
|  |  |  |  |  | hsa-miR-326 | hsa-miR-181c |
|  |  |  |  |  | hsa-miR-34a | hsa-miR-181d |
|  |  |  |  |  | hsa-miR-34c-5p | hsa-miR-186 |
|  |  |  |  |  | hsa-miR-370 | hsa-miR-194 |
|  |  |  |  |  | hsa-miR-377 | hsa-miR-200a |
|  |  |  |  |  | hsa-miR-500 | hsa-miR-200b |
|  |  |  |  |  | hsa-miR-532-3p | hsa-miR-200c |
|  |  |  |  |  | hsa-miR-543 | hsa-miR-203 |
|  |  |  |  |  | hsa-miR-548d-3p | hsa-miR-220b |
|  |  |  |  |  | hsa-miR-582-3p | hsa-miR-223 |
|  |  |  |  |  | hsa-miR-647 | hsa-miR-23a |
|  |  |  |  |  | hsa-miR-648 | hsa-miR-23b |
|  |  |  |  |  | hsa-miR-891b | hsa-miR-27a |
|  |  |  |  |  | hsa-miR-9 | hsa-miR-27b |
|  |  |  |  |  |  | hsa-miR-299-5p |
|  |  |  |  |  |  | hsa-miR-301 |
|  |  |  |  |  |  | hsa-miR-301 |
|  |  |  |  |  |  | hsa-miR-301b |
|  |  |  |  |  |  | hsa-miR-302a |
|  |  |  |  |  |  | hsa-miR-302b |
|  |  |  |  |  |  | hsa-miR-302c |
|  |  |  |  |  |  | hsa-miR-302d |
|  |  |  |  |  |  | hsa-miR-329 |
|  |  |  |  |  |  | hsa-miR-330-3p |
|  |  |  |  |  |  | hsa-miR-340 |
|  |  |  |  |  |  | hsa-miR-362-3p |
|  |  |  |  |  |  | hsa-miR-372 |
|  |  |  |  |  |  | hsa-miR-373 |
|  |  |  |  |  |  | hsa-miR-429 |
|  |  |  |  |  |  | hsa-miR-454 |
|  |  |  |  |  |  | hsa-miR-495 |
|  |  |  |  |  |  | hsa-miR-501-3p |
|  |  |  |  |  |  | hsa-miR-509-3-5p |
|  |  |  |  |  |  | hsa-miR-509-5p |
|  |  |  |  |  |  | hsa-miR-518a-5p |
|  |  |  |  |  |  | hsa-miR-520a-3p |
|  |  |  |  |  |  | hsa-miR-520b |
|  |  |  |  |  |  | hsa-miR-520c-3p |
|  |  |  |  |  |  | hsa-miR-520d-3p |
|  |  |  |  |  |  | hsa-miR-520d-5p |
|  |  |  |  |  |  | hsa-miR-520e |
|  |  |  |  |  |  | hsa-miR-520f |
|  |  |  |  |  |  | hsa-miR-524-5p |
|  |  |  |  |  |  | hsa-miR-527 |
|  |  |  |  |  |  | hsa-miR-543 |
|  |  |  |  |  |  | hsa-miR-545 |
|  |  |  |  |  |  | hsa-miR-549 |
|  |  |  |  |  |  | hsa-miR-551a |
|  |  |  |  |  |  | hsa-miR-556-5p |
|  |  |  |  |  |  | hsa-miR-606 |
|  |  |  |  |  |  | hsa-miR-607 |
|  |  |  |  |  |  | hsa-miR-617 |
|  |  |  |  |  |  | hsa-miR-620 |
|  |  |  |  |  |  | hsa-miR-656 |
|  |  |  |  |  |  | hsa-miR-802 |
|  |  |  |  |  |  | hsa-miR-9 |
